# Publication Counts in Context: Normalization Using Query and Reference Terms in PubMed

**DOI:** 10.2196/60616

**Published:** 2025-02-03

**Authors:** Julian Varghese, Lucas Bickmann, Timo Strünker, Nina Neuhaus, Frank Tüttelmann, Sarah Sandmann

**Affiliations:** 1 Institute of Medical Informatics University of Münster Münster Germany; 2 Centre of Reproductive Medicine and Andrology University Hospital Münster University of Münster Münster Germany; 3 Centre of Medical Genetics University of Münster Münster Germany

**Keywords:** publication database, science communication, citation, H-index, normalization, publication, trend, scientometrics, scholarly

## Abstract

This article discusses the extensive use of publication counts as indicators of trends in the scientific activities of individual researchers, research groups, and entire disciplines. However, with the growing number of articles in general, these counts might produce false impressions among scientists. We propose a straightforward yet effective normalization method, which enables further context of publication counts by using a query and a reference term. Additionally, an open access implementation is readily available on the PubMed Normalization website.

## Problem Definition

**Figure 1:**
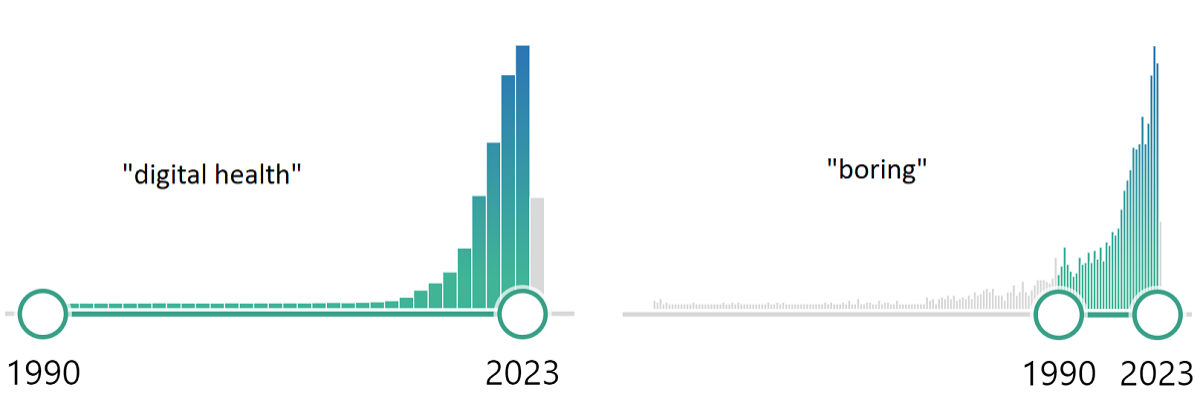
Original plot of PubMed counts per year, executed on May 2, 2024. Search terms: “digital health” (left) and “boring” (right).

PubMed is a widely used literature database that provides access to a vast repository of biomedical and life sciences literature. Publication counts on PubMed can be visualized through simple plots that display the number of publications over time, providing an easy way to track trends and patterns in scientific output. Publication counts over time are often used in science communication, research applications, original articles, or reviews to illustrate trends in scientific activity in a particular field [1-4]. A search on PubMed readily generates corresponding publication counts from year to year (Figure 1). To indicate growing scientific output of individuals or even joint consortia on a particular topic, the search can be further specified by author names, research sites, or affiliations. This yearly summary plot can and probably will be used as a valuable overview for (1) individual researchers or research groups to claim the success of recent research activities and (2) for policy makers or research funding organizations for initial decision-making or judging on research applications.

However, apart from its usefulness and simplicity, the plot can generate a false impression, which the authors of this viewpoint paper would like to stress on as they are frequently experiencing this type of illustration in presentations or publications without mentioning 2 important issues.

First, it does not take into account the fact that the overall number of publications have increased disproportionally. Figure 1 illustrates the problem for 2 query terms “digital health” and “boring” with a similar progression of publication counts.

**Figure 2:**
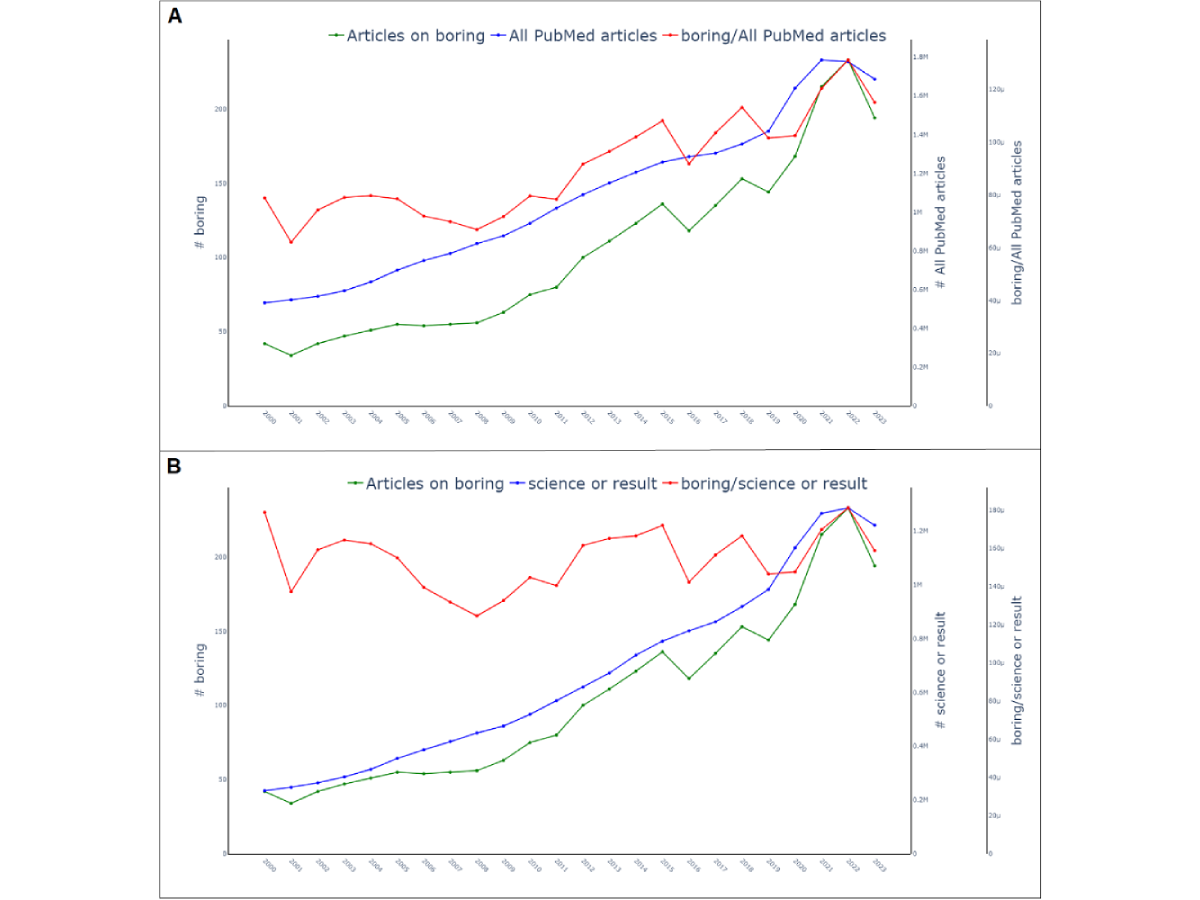
Article counts for the query term boring. (A) A simple correction when dividing the number of articles per year by the number all PubMed articles per year still shows an increase (red graph). (B) Correction applied by using a reference term, for example, science" or result. See Multimedia Appendix 1 for larger-resolution images.

The second issue is the potential textual growth per article over time, for example, by increasing the abstract’s length or further searchable metadata per article. For instance, there is evidence that the abstract length of articles has increased over time in Cochrane Reviews [5], and some articles may even exceed the abstract length that is allowed by a medical journal [6]. We are dealing with an increase in not only article counts but also textual growth per article, illustrated in Figure 2A by using the search term *boring*. Textual growth per article increases the base probability of a query term to be found and thus artificially increases publication counts per year.

While the available advanced search functions on PubMed allow for more targeted approaches, they do not provide a normalization of publication counts and their visualization, which is essential for accurately interpreting trends in the context of overall publication growth over time.

## Pragmatic Solutions

Certainly, there are numerous correction measures involving advanced statistical and semantic analyses, which could add deeper insights for a more differentiated way of interpreting search results. Moreover, commercial tools are available, such as SciVal by Elsevier or Web of Science and Insight by Clarivate, which use more comprehensive datasets from larger literature databases and citation data to generate individual research portfolios or analyze publication trends. Here, we propose a noncommercial, pragmatic approach, which does not overwhelm the user with statistical details but retains the simplicity of the original approach as illustrated in Figure 1.

This issue of the overall increasing number of publications can be tackled by dividing the counts by the number of all PubMed publications within the corresponding year. This straightforward correction is already applied in implementations such as the web-based tool PubMed by Year [7]*,* which also offers the user to query and compare publication counts of other search queries*.* This does, however, not solve the issue regarding textual growth per article. Figure 2A shows that counts over time for the query term *boring* are increasing despite the aforementioned correction.

To overcome both issues, we suggest calculating and visualizing a normalization ratio, in which the counts of a query term are divided by the counts of a reference term. Both the query and the reference terms can be specified by the user, for instance by using a basic set of frequently used unspecific terms, such as *research*, *science*, or *results*. These reference terms provide a more robust correction because they are also impacted by the 2 aforementioned issues, leading to an adjustment where errors cancel out. Users have the flexibility to modify the reference term to better suit their specific needs or areas of interest.

**Figure 3:**
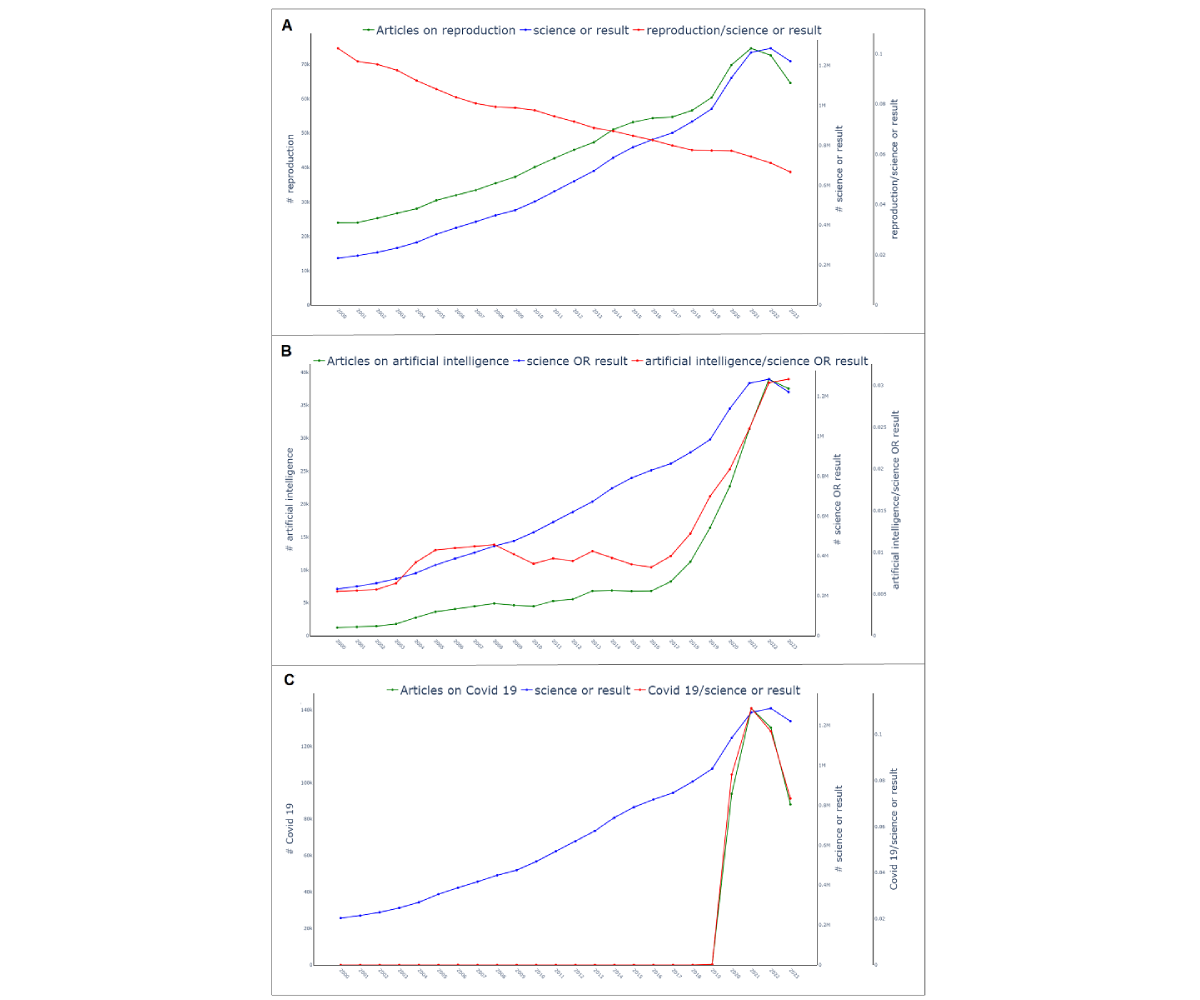
While the query terms reproduction (A) and artificial intelligence (B) both show an increase in the number of publications per year (green line), the normalization ratios (red line) reveal a steady decrease for reproduction (A). For artificial intelligence (B), the red line indicates steady growth since 2016. After 2022, the growth rate slowed down. For the query term Covid 19 (C) the green and the red lines are almost perfectly aligned, indicating a steep increase followed by a steep decrease in the number of absolute publications and relative publication counts. See Multimedia Appendix 2 for larger-resolution images.

Figure 2B shows an example plot that presents the normalization ratio. The search term *boring* now shows a steady publication count with minor variations per year. Figure 3 provides complementary examples for the search terms *Reproduction*, showing an established field with a decreasing normalization ratio; *Artificial Intelligence*, showing a continuously growing field; and *COVID-19*, showing a short-term steep increase followed by a decrease as the result of a pandemic.

A ready-to-use tool that calculates and visualizes the corrective normalization ratio for any user-specified PubMed query term is provided on the PubMed Normalization website or as a Docker container with open-source code [8].

## Discussion

PubMed search counts can generate a valuable overview of research trends but should be treated with caution as they represent nonnormalized and therefore potentially misleading output. The main advantage is its simplicity regarding usage and initial interpretation. For more insightful interpretation, we have pointed out 2 issues, namely the increasing article count and the textual growth per article. With this viewpoint paper, we would like to raise awareness when interpreting such publication counts. The rising number of articles published each year can lead to an overwhelming amount of data, making it difficult to identify meaningful trends without further statistical analyses and domain-specific knowledge.

We suggest adding more context to publication counts by using a normalization ratio graph that uses a reference term to account for both aforementioned issues. All parameters can be specified by the user to account for subject-related queries or reference terms. Moreover, PubMed’s advanced search function is integrated as well, as our ready-to-use demonstrator supports the original PubMed queries as an input. It should be noted that this analysis cannot replace deeper analyses that address well-known drawbacks from literature searches, such as publication and accessibility bias, language bias, field-specific variations regarding publishing practices, or query and reference term selection. Nevertheless, we believe that a combined graph provides the same simplicity as the naïve search and provides more robust results when presenting publication counts and comparing them to the general research output.

In our search for related bibliometric analyses tools on the web, we have found numerous and highly valuable commercial tools such as SciVal by Elsevier or InCites by Clarivate, or open-source tools like bibliometrix [9] and PubMed by Year [7]. While these come with extensive analyses features to analyze individual or network-based research output and collaboration patterns, not only on PubMed but also on many different literature databases, we could not find a free and readily available web-based tool with a specific way to normalize and instantly visualize PubMed counts against a reference term. For quick demonstration, we have provided an exemplary implementation, which is accessible on a sustained web-based infrastructure. We encourage the scientific community to view the proposed method as a foundational tool, designed to address the key limitations of current article count presentations. While this approach offers a basic correction, we advocate for further customization to meet the specific demands of diverse research landscapes.

## Appendix

Multimedia Appendix 1 Article counts for the query term boring. (A) A simple correction when dividing the number of articles per year by the number all PubMed articles per year still shows an increase (red line). (B) Correction applied by using a reference term, for example, science or result.

Multimedia Appendix 2 While the query terms reproduction (A) and artificial intelligence (B) both show an increase in publications per year (green lines), the normalization ratios (red lines) reveal a steady decrease for reproduction (A). For artificial intelligence (B), the red line indicates steady growth since 2016. After 2022, the growth rate slowed down. For the query term Covid 19 (C) the green and the red lines are almost perfectly aligned indicating a steep increase followed by a steep decrease of absolute publication and relative publication counts.
