# Supplementary figures and images for: Publication Counts in Context: Normalization Using Query and Reference Terms in PubMed
Source: J Med Internet Res. 2025 Feb 3;27:e60616. doi: 10.2196/60616 (PMC11833257; doi:10.2196/60616)

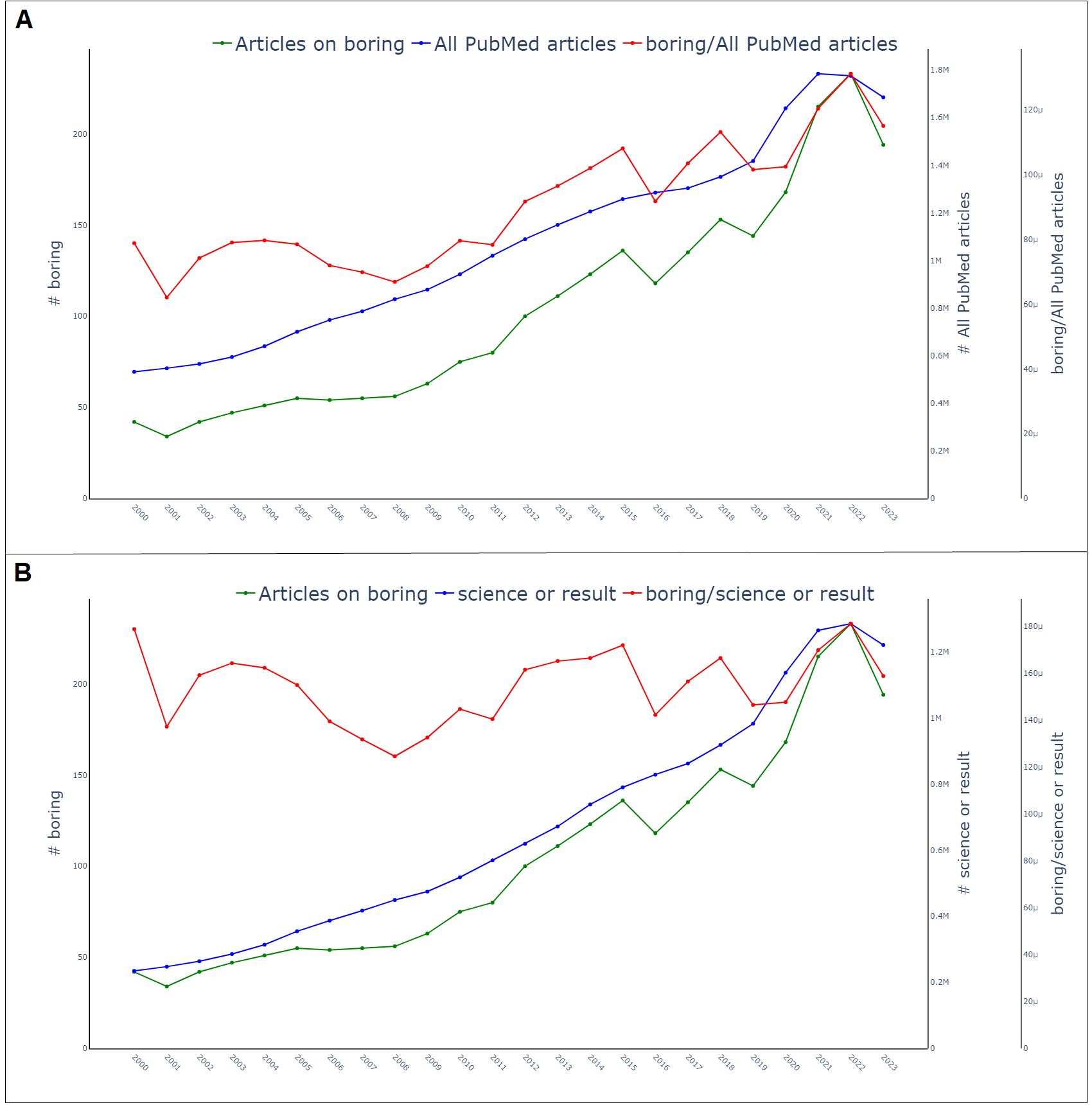

Supplement: Multimedia Appendix 1 [file jmir_v27i1e60616_app1.png]

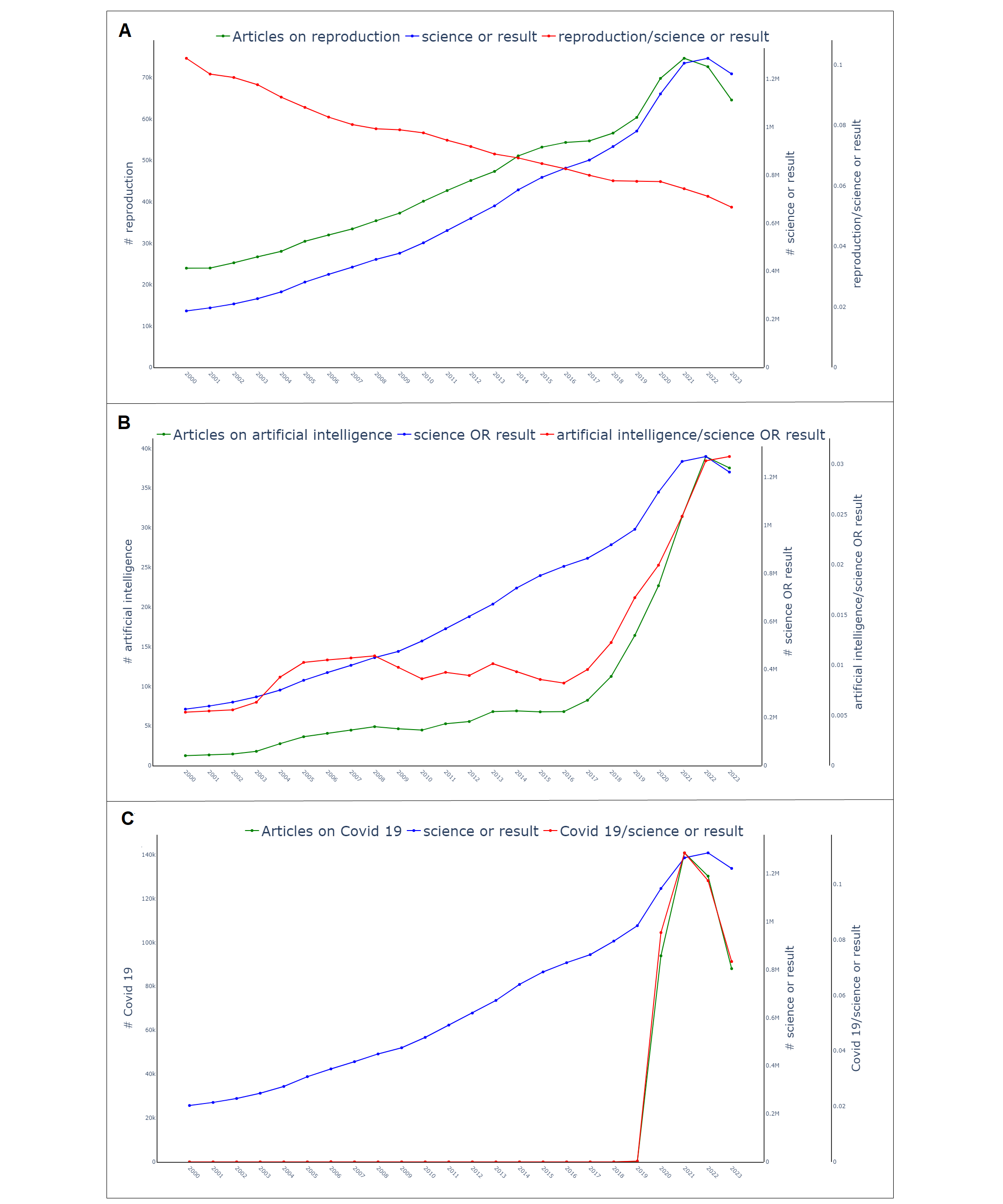

Supplement: Multimedia Appendix 2 [file jmir_v27i1e60616_app2.png]
